# Mechanical Characterization of the Tensile Properties of Glass Fiber and Its Reinforced Polymer (GFRP) Composite under Varying Strain Rates and Temperatures

**DOI:** 10.3390/polym8050196

**Published:** 2016-05-19

**Authors:** Yunfu Ou, Deju Zhu, Huaian Zhang, Liang Huang, Yiming Yao, Gaosheng Li, Barzin Mobasher

**Affiliations:** 1 College of Civil Engineering, Hunan University, Changsha 410082, China; ouyunfu@hnu.edu.cn (Y.O.); zhanghuaianjs@outlook.com (H.Z.); ligaosheng@hnu.edu.cn (G.L.); 2 School of Sustainable Engineering and Built Environment, Arizona State University, Tempe, AZ 85287, USA; Yiming.Yao@asu.edu (Y.Y.); Barzin@asu.edu (B.M.)

**Keywords:** polymer-matrix composites (PMCs), mechanical properties, stress/strain curves, deformation, statistics

## Abstract

Unidirectional glass fiber reinforced polymer (GFRP) is tested at four initial strain rates (25, 50, 100 and 200 s^−1^) and six temperatures (−25, 0, 25, 50, 75 and 100 °C) on a servo-hydraulic high-rate testing system to investigate any possible effects on their mechanical properties and failure patterns. Meanwhile, for the sake of illuminating strain rate and temperature effect mechanisms, glass yarn samples were complementally tested at four different strain rates (40, 80, 120 and 160 s^−1^) and varying temperatures (25, 50, 75 and 100 °C) utilizing an Instron drop-weight impact system. In addition, quasi-static properties of GFRP and glass yarn are supplemented as references. The stress–strain responses at varying strain rates and elevated temperatures are discussed. A Weibull statistics model is used to quantify the degree of variability in tensile strength and to obtain Weibull parameters for engineering applications.

## 1. Introduction

Fiber reinforced polymer (FRP) composites have many merits, such as high stiffness/weight and strength/weight ratios, advanced fatigue and corrosion resistances, *etc.*, providing significant functional and economic benefits, ranging from strength enhancement and weight reduction to durability features. With decreasing manufacturing costs, recently, they have won the attention of engineers involved in the construction of civil structures [1]. Structural elements reinforced with FRP, however, might be subjected to dynamic loadings, such as wind loads, earthquake loads, explosions, *etc.* and vary temperature conditions during their service life. Under such conditions, the mechanical properties of FRP involving Young’s Modulus, tensile strength, toughness, *etc.* may suffer great changes [2,3,4,5,6]. Therefore, the investigation of the mechanical properties of FRP composites under dynamic loadings and different temperatures is essential to design the structures with this kind of materials.

A variety of testing techniques and procedures have been developed for gaining insight into the dynamic responses of materials under different strain rates and different methods are suited for different ranges of strain rate. The conventional screw drive load frame [7] is used for quasi-static loading of coupons at a constant strain rate. In the medium strain-rate region for strain rates up to approximately 200 s^−1^, servo-hydraulic high speed machines [8] or drop-weight impact systems [9] can be used. The most widely used technique for obtaining direct determination of material properties at strain rates between 200 and 10^4^ s^−1^ is the split Hopkinson bar, which was first introduced by Kolsky [10]. With the help of those machines, rate dependent data of FRP composites can be obtained.

The tensile behaviors of carbon fiber-reinforced polymers (CFRP) under different strain rates were studied by several researchers [7,11,12,13]. It was reported that the tensile properties of CFRP are strain rate dependent, while the average transverse modulus is independent of strain rate. Koerber *et al.* [14] found that shear modulus and shear behavior of the carbon-epoxy material system IM7-8552 are dependent of strain rate. Specifically, when the loading case varied from quasi-static to dynamic, the in-plane shear modulus of elasticity, yield strength and pure failure strength increased by 25%, 88% and 42%, respectively. Strain rate effects on aramid fiber-reinforced polymers (AFRP) were described in literature [15]. It showed that both the Young’s modulus and tensile strength increase at the impact loading rate, while the failure strain decreases slightly. As the most widely used FRP composites in civil engineering due to lower cost of production, GFRP, especially its behaviors under dynamic loadings has also drawn much attention. Barre *et al.* [16] determined the tensile behavior of GFRP using a drop-weight dynamic testing machine. The results revealed that dynamic elastic modulus and strength tend to increase with increasing strain rate. Shokrieh *et al.* [17] studied the tensile properties of unidirectional GFRP composites at quasi-static and intermediate strain rates of 0.001–100 s^−1^ by means of a servo-hydraulic testing apparatus. A significant increase of the tensile strength was observed with increasing strain rate. Ochola *et al.* [18] investigated the strain rate sensitivity of GFRP at strain rates of 10^−3^ and 450 s^−1^. The experimental results reported that the dynamic material strength of GFRP increases with increasing strain rates.

Temperature effects on GFRP are another important issue that needs further investigation in order to adopt the use of such composites in strengthening structural reinforced concrete (RC) members and their connections when exposed to elevated temperatures and harsh environment. Hawileh *et al.* [19] experimentally investigated the variation of mechanical properties in terms of the elastic modulus and tensile strength of composite glass (C), composite glass (G) sheets and their hybrid combinations (CG) when exposed to different temperatures, ranging from 25 to 300 °C. Results showed that the elastic modulus of the C, G and CG at 250 °C was reduced by nearly 28%, 26% and 9%, respectively, and their tensile strength at the same temperature was reduced by about 42%, 31% and 35%, respectively, all as compared to room temperature values. Robert *et al.* [20] evaluated the variation of mechanical properties of sand-coated GFRP reinforcing bars subjected to low temperatures (ranging from 0 to −100 °C) and elevated temperatures (ranging from 23 to 315 °C) and discovered that low temperature has a positive influence on the strength of composites, while at very high temperatures, around about the glass-transition temperatures of the polymer matrix, the mechanical properties, especially the stiffness and the strength of the composites are decreased significantly. Reis *et al.* [6] conducted tensile tests on GFRP at different strain rates and temperatures and found that strain rate greatly affects the ultimate tensile strength, and the Young’s modulus is almost insensitive to it while temperature only influences the modulus. GFRP samples with single yarn were tested at different strain rates from quasi-static up to 160 s^−1^ and temperatures from −25 to 100 °C by Ou and Zhu [9] to investigate any possible effects on their mechanical properties and failure patterns. The results showed that the tensile strength, maximum strain and toughness increase with increasing strain rates at room temperature, and the Young’s modulus, tensile strength and toughness decrease with increasing temperatures at the strain rate of 40 s^−1^.

Although many efforts have been carried out on the strain rate and temperature effects on the mechanical properties of GFRP, very limited information is available on intermediate strain rate ranging from 25 to 200 s^−1^, and only few experimental results are accessible on the dynamic tensile behavior of GFRP at elevated temperatures and harsh environment. Furthermore, as the main load-bearing body of GFRP, glass fibers play a significant role in GFRP subjected to an impact load. Therefore, revealing the variation of tensile properties of fibers at different strain rates is the key to gaining a deep insight into the mechanical behavior of GFRP under impact loads and then optimizing structural design.

The primary objective of this study is to investigate the effects of intermediate strain rates and temperatures on the mechanical properties of glass fiber bundle (yarn) and unidirectional GFRP laminate samples. In the next section, the experimental procedure and test results are presented and discussed. Then, comparison of the obtained values of tensile strength of GFRP specimens at different strain rates with the values found in literature is displayed. In the fourth section, the results of Weibull statistical analysis based on experimental results are discussed.

## 2. Experimental Program

### 2.1. Testing Materials

**Figure 1:**
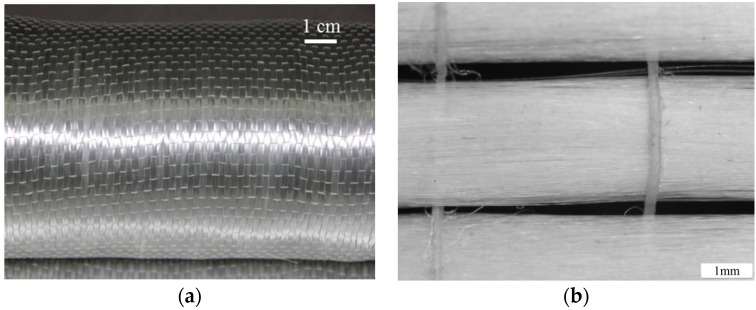
(**a**) Woven structure and (**b**) microscopy image of unidirectional glass fiber fabric.

The glass unidirectional fiber fabric made by Nanjing Hitech Composites Co., Ltd. (Nanjing, China) was used in this study, which had been fabricated with individual yarns (5.3 yarns/cm), as shown in Figure 1a. The individual yarns in the fabric consist of thousands of filaments as shown in Figure 1b. The cross-sectional area of single glass yarn were calculated as 0.473 mm^2^ by taking into account the linear density of the material and dividing it by its bulk density, following the same method in the reference [21]. Epoxy resin provided by Hunan Good-Bond Construction Technic Development Co., Ltd. (Changsha, China) was utilized as the matrix. The physical and mechanical properties of GFRP components, *i.e.*, the glass yarn and epoxy resin, were listed in Table 1.

### 2.2. Specimen Fabrication and Test Procedures

**Figure 2:**
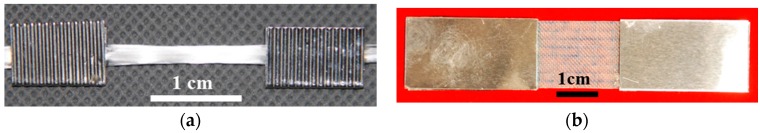
Prepared samples of (**a**) glass yarn and (**b**) GFRP.

Individual yarn was extracted carefully from the glass fabric for single yarn tests. In order to reduce stress concentration and to improve the load transfer mechanism between the specimens and steel wedges, thin aluminum sheets (10 mm × 5 mm × 0.5 mm) were roughhewed by toothed steel plate and glued at both ends of the single yarn specimens using the same epoxy. When the epoxy was fully cured, the final glass yarn specimen was constructed by cutting off the redundant length at both sides using an electric scissor as shown in Figure 2a.

GFRP were manufactured by Vacuum Assisted Resin Transfer Molding (VARTM) [22], thus thin laminates composed of a single ply of glass fabric with epoxy resin were fabricated, with a thickness of nearly 0.52 mm, after curing 24 h at room temperature. Due to the laminate heterogeneity, the specimens should be large enough to have mechanical properties representative of the material. Considering the limitation of grip, specimens were cut along the fiber direction from the laminate with a dimension of 105 mm × 22 mm (L × W). Two aluminium sheets of 40 mm long, 22 mm wide and 0.3 mm thick were glued on the end of specimens to avoid stress concentration. When the epoxy was fully cured, the typical test specimens were shown in Figure 2b. The gauge lengths of specimen are 25.0 mm, and there are 8 glass fiber yarns in the width with a volume fraction of 34.3%.

**Figure 3:**
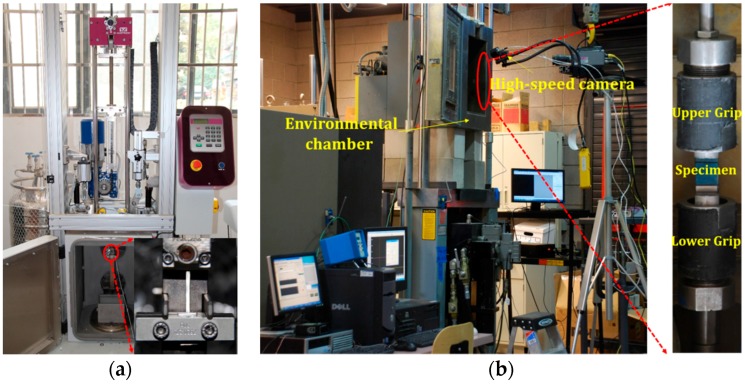
Experimental setup: (**a**) Instron drop-weight impact system; (**b**) Mechanical Testing & Simulation (MTS) servo-hydraulic high speed testing machine.

Dynamic tensile tests of glass yarn under various strain rates and temperatures were conducted on a state-of-the-art drop-weight impact system (Instron, CEAST9340, Shanghai, China) as shown in Figure 3a. The drop height ranges from 0.03 to 1.10 m with corresponding impact velocity ranging from 0.77 to 4.65 m/s. The maximum load application is 90 kN with a maximum potential energy of 405 J. The required impact velocities can be achieved by releasing the impactor with a weight of 52 N from predetermined heights. The impact force induced by the free fall weight was measured by piezoelectric force transducer with a capacity of 2.2 kN, and the force, deformation and energy *versus* time were recorded simultaneously by a high speed data acquisition system at a sampling rate of 1 MHz with 14-bit resolution. In addition, the CEAST Visual Impact software (INSTRON (China) Co., Ltd., Shanghai, China) is used for system management and data processing (including the strain calculation). An environmental chamber can be heated by electric resistance wire and cooled by liquid nitrogen, giving operating temperatures ranging from −50 to 100 °C. In this work, four different impact velocities (1, 2, 3 and 4 m/s) were chosen to obtain the strain rates of 40, 80, 120 and 160 s^−1^ for specimens at room temperature (25 °C). In addition, four different temperatures, *i.e.*, 25, 50, 75 and 100 °C were selected for the study of temperature effect under an impact velocity of 1 m/s (strain rate = 40 s^−1^). Ten samples were tested for each strain rate and temperature.

In addition, dynamic tensile tests of GFRP specimens under various strain rates and temperatures were performed using an MTS high rate servo-hydraulic testing machine with an environmental chamber in Arizona State University (as shown in Figure 3b) due to the limited force measurement capacity of the drop-weight impact system. The speed of the stroke is controlled by the opening and closing of the servo-valve of hydraulic supply. By manually turning the servo-valve, the rate of flow of hydraulic fluid can be controlled, thus a desired stroke speed can be obtained. In our test, the valve was opened and the stroke accelerated until it reached a constant predetermined velocity, then the test specimens were mounted between upper and lower grips. These grips have serrated surfaces to effectively clamp specimens and prevent any slippage during the loading process of tests. According to the calibration records, the stroke can reach a maximum speed of 14 m/s with a load capacity of 25 kN [23]. Operating temperatures ranging from −60 to 200 °C were achieved by liquid nitrogen and electric resistance wire of the environmental chamber, with a built-in fan to ensure the uniform distribution of temperature in the chamber. At room temperature (25 °C), dynamic tensile tests were conducted at four loading velocities of 0.625, 1.25, 2.5 and 5 m/s. The initial strain rates are defined as the ratio of the applied velocity to the specimen’s gauge length, namely 25, 50, 100 and 200 s^−1^, respectively. At the initial strain rate of 25 s^−1^, six different temperatures (−25, 0, 25, 50, 75 and 100 °C) were selected for temperature effect testing. The automatically controlled environmental chamber was set to the required temperature and maintained for 30 min to guarantee that the test specimens reached a uniform temperature. Ten replicates were tested for each condition to reduce the influence of random error. A high-speed digital acquisition card (TRIO Test & Measurement, Adelaide, Australia) was used to collect force and displacement data at a sampling rate of 500 kHz. A Phantom v7.3 high speed digital camera (Vision Research Phantom, Wayne, NJ, USA) was used to capture the deformation and failure behavior of GFRP specimens with a sampling rate of 20,000 fps.

Quasi-static testing was performed on an MTS load frame (C43.304) (MTS Systems (China) Co., Ltd., Shanghai, China) [24] with a load capacity of 30 kN and maximum sampling rate of 1000 Hz. In this work, two load cells with 1 and 30 kN capacities were used for force measurement of glass yarn and GFRP, respectively, with a sampling rate of 20 Hz, and the loading velocity was set to be 2.5 mm/min. The corresponding strain rate is 1/600 s^−1^ by dividing the velocity with gauge length. An extensometer was used for displacement measurement of GFRP, while, for glass yarn, the displacement recorded by MTS load frame is very close to that measured by the extensometer with an error less than 2%, so it is reasonable to use the crosshead measurement as the deformation of glass yarn for strain calculation.

## 3. Results and Discussion

### 3.1. Strain Rate Effect on Tensile Properties

**Figure 4:**
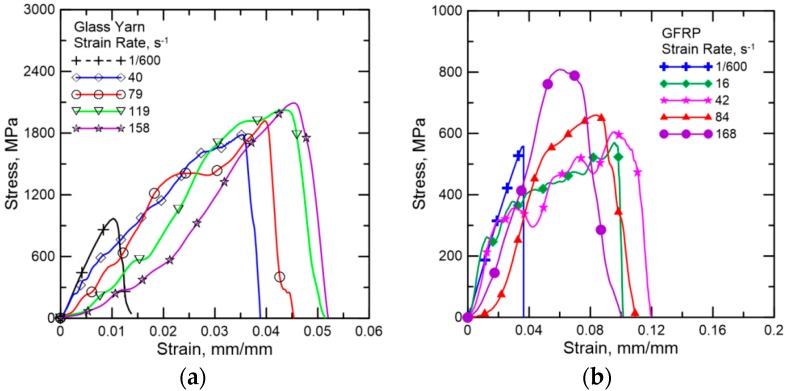
Representative stress–strain responses of (**a**) glass yarn at initial strain rates of 1/600, 40, 80, 120, 160 s^−1^ and (**b**) GFRP at initial strain rates of 1/600, 25, 50, 100, 200 s^−1^.

**Figure 5:**
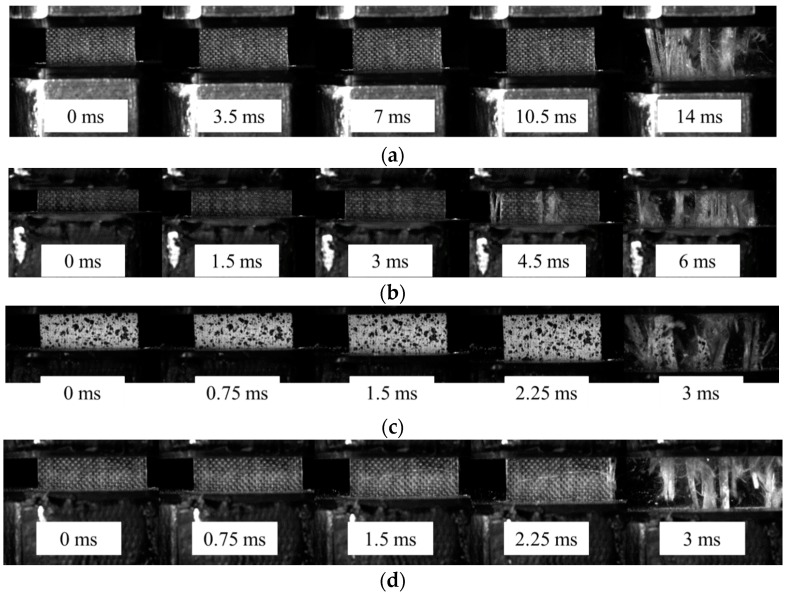
Failure behaviors of GFRP at different strain rates: (**a**) 25 s^−1^; (**b**) 50 s^−1^; (**c**) 100 s^−1^ and (**d**) 200 s^−1^.

Figure 4a show the stress *vs.* strain curves of glass yarn samples at different strain rates. The high-frequency oscillations in the stress curves obtained under dynamic loadings were caused by the system ringing phenomenon [25,26]. In addition, the number of oscillations in the curves decreases with increasing impact velocities, since the impact duration is much shorter at higher loading rates. Figure 4b shows the stress *vs.* strain curves of GFRP samples at different strain rates. Note that the actual strain rates during dynamic testing was remarkably different from the initial strain rates, which is attributed to the fact that the reaction force of the test specimen slowed down the stroke, especially at higher loading speeds when the hydraulic pressures were relative lower. In the subsequent discussion of this paper, the initial strain rates will be used to facilitate analysis. Meanwhile, it is also worth noting that the curves were similar with the strain–stress curve of mild steel because the stress fluctuated after a small elastic deformation until the final failure at 25 and 50 s^−1^. This is because the yarns of GFRP samples do not fail simultaneously during the loading, as shown in Figure 5. However, the fluctuation in stress reduced at a higher strain rate, and it disappeared when the strain rate reached 200 s^−1^.

**Figure 6:**
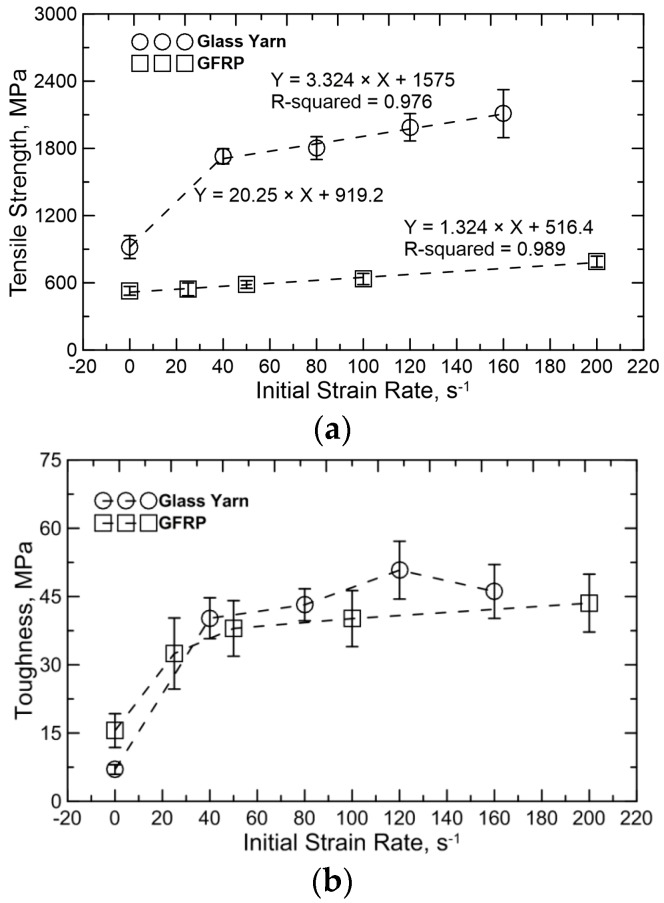
Strain rate effect on tensile properties: (**a**) tensile strength and (**b**) toughness.

The effects on mechanical properties of GFRP can be summarized based on the stress–strain curves under different strain rates. Figure 6 shows the dependence of the dynamic tensile properties of glass yarn and GFRP, defined in terms of tensile strength and toughness, on the varying initial strain rates, respectively. The toughness is calculated as the area under stress-strain curves, given by:

(1) $$U_{T}=\frac{Energy}{Volume}=\int_{0}^{\varepsilon_{f}}\sigma d\varepsilon$$

where the term $U_{T}$ represents toughness; ε is strain; $\varepsilon_{f}$ is the maximum strain and σ is stress. There is an apparent dependence on the dynamic tensile properties of glass yarn and GFRP on the strain rate. For the glass yarn, tensile strength and toughness increase significantly during a transition from quasi-static loading (1/600 s^−1^) to dynamic loading (40 s^−1^). In addition, tensile strength maintains growth with increasing strain rate, while toughness abruptly decreases at the strain rate of 160 s^−1^ due to the diminution of strain. Specifically, tensile strength and toughness increase as much as 88.0% and 474.3% from 919 ± 102 MPa and 7.0 ± 1.1 MPa to 1729 ± 67 MPa and 40.2 ± 4.5 MPa, respectively, when the strain rate increases from 1/600 to 40 s^−1^. After that, tensile strength goes on to increase nearly 22.0% when the strain rate increases from 40 to 160 s^−1^, while toughness increases about 26.4% over a strain rate range of 40–120 s^−1^ and then nearly drops by 10.2% between 120 and 160 s^−1^. The initial significant enhancement in strength and toughness can be explained as: the friction between adjacent fibers always plays an important role due to random and misaligned breakage of filaments in the yarn under tension and the contact force among adjacent filaments increases with increasing strain rate, resulting in the increase of dynamic frictional force (friction coefficient is assumed to be constant). Therefore, tensile strength and energy-consumption are improved noticeably.

For GFRP, the tensile strength linearly increases over the strain rate range of 1/600–200 s^−1^, while the variation of its toughness is similar to the tensile strength of glass yarn. Concretely speaking, tensile strength increases nearly 49.1% when the strain rate increases from 1/600 to 200 s^−1^. By contrast, toughness increases remarkably from 15.5 ± 3.7 MP a at a strain rate of 1/600 s^−1^ to 32.5 ± 7.8 MPa at 25 s^−1^, enhanced by 109.7%, and then increases about 34.2% over the strain rate range of 25–200 s^−1^. Those kinds of strengthening and toughening mechanisms are different from glass yarn and could be described as: (i) there is not enough time to initiate internal defects in the material at higher strain rates. Therefore, more energy is needed for damage initiation and propagation, which leads to a higher level of tensile strength under high strain rates, and (ii) more damage is involved with increasing strain rate.

**Figure 7:**
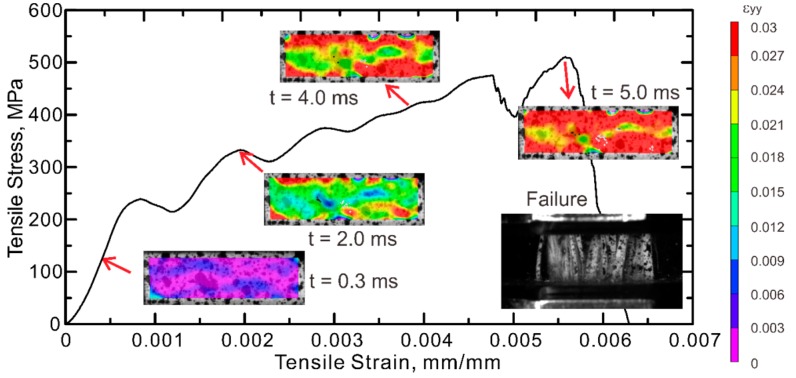
2D contour of ε_yy_ and stress–strain response for a GFRP specimen tested at the strain rate of 25 s^−1^ obtained by digital image correlation (DIC).

**Figure 8:**
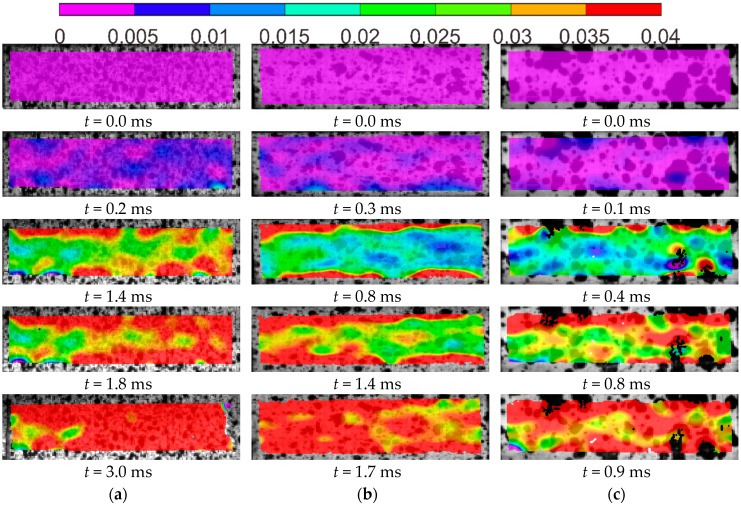
Normal strain distribution of GFRP adherent at different loading rates: (**a**) 50 s^−1^; (**b**) 100 s^−1^; and (**c**) 200 s^−1^.

In order to further study the tensile behavior and failure pattern of GFRP, digital image correlation (DIC) analysis was performed on the specimens tested under room temperature. The stress–strain curve of a GFRP tested at 25 s^−1^ together with the longitudinal strain (ε_yy_) distributions measured by DIC are shown in Figure 7. A color code with purple representing the lowest strain values and red at 3.0% strain is used, denoting the level of strains. The time and stage of stress–strain behavior associated with each step of the strain distributions are indicated. The damage evolution shows that, during the linear elastic stage of the test (*t* = 0.3 ms), a relatively uniform strain distribution is obtained. As the load increases (*t* = 2.0 ms), tensile strain starts to localize near the top and bottom edges of area of interest (AOI), which is represented by the red zone. This may imply the stress concentration due to the clamps and associate with the non-linear behavior observed in the stress-strain curve. The inhomogeneity of the strain fields may be attributed to multiple aspects such as the presence of random defects, variability in the stiffness of basalt yarns and potential eccentric loads. The localized zones continue to grow with increasing load (*t* = 4.0 ms) and ultimately spread out over the entire AOI prior to failure (*t* = 5.0 ms). Non-uniform strain fields are also observed in the specimens tested at other strain rates, as shown in Figure 8.

**Figure 9:**
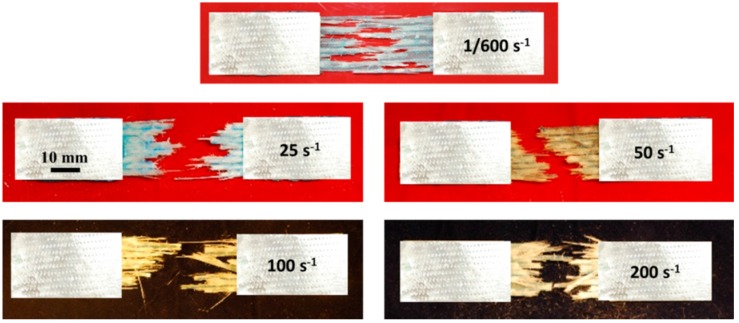
Fracture morphologies of GFRP under different strain rates.

Figure 9 compares the failure morphologies of the composite specimens after tension under different strain rates, which clearly shows a remarkable difference in the fracture areas. It seems from the fracture surface of relative low strain rate (1/600 s^−1^), the damage area was limited to a small region; however, under higher strain rate (200 s^−1^), the damage path covers the entire gauge section, indicating more materials of the specimen are involved in energy dissipation. This phenomenon was also observed by Shokrieh [17] in a relevant experiment.

### 3.2. Temperature Effect on Tensile Properties

**Figure 10:**
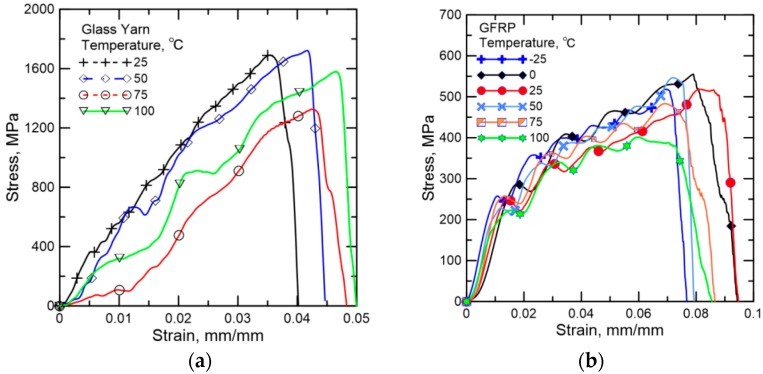
Representative stress–strain responses of (**a**) glass yarn at temperatures of 25, 50, 75, 100 °C and (**b**) GFRP at temperatures of −25, 0, 25, 50, 75, 100 °C.

**Figure 11:**
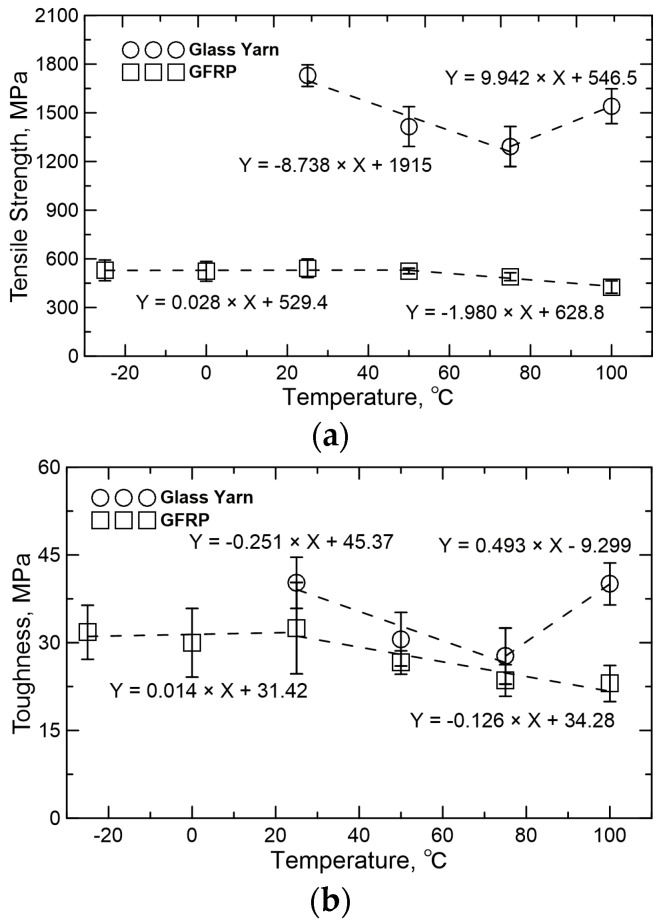
Temperature effect on tensile properties: (**a**) tensile strength and (**b**) toughness.

Figure 10 shows the experimental stress-strain responses of glass yarn and GFRP under different temperatures with the same strain rate of 25 s^−1^. The effect of temperature on tensile strength and toughness in terms of average values and standard deviations are summarized in Figure 11. For glass yarn, tensile strength and toughness decrease nearly 25.3% and 31.1% when temperature increases from 25 to 75 °C. This is because the fracture process of fibers is generally accompanied by the damage of chemical bond, intermolecular slip and the weakening of van der Waals force, *etc.* Heating will undoubtedly weaken the chemical bond and van der Waals force and then lower the tensile strength of fibers. Furthermore, maximum strain over temperature range of 25–75 °C has no obvious change, so toughness also decreases. However, when heated to 100 °C, tensile strength of glass yarn rebounds 19.2% at the base of value at 75 °C, mainly due to the fact that the frictional coefficient of the fiber surface increases because of volatilization of sizing and water at 100 °C, resulting in the increase of frictional force between fibers. At the same time, toughness gets a sharp increase (about 44.5%), which could be attributed to the obvious increases in both tensile strength and maximum strain.

**Figure 12:**
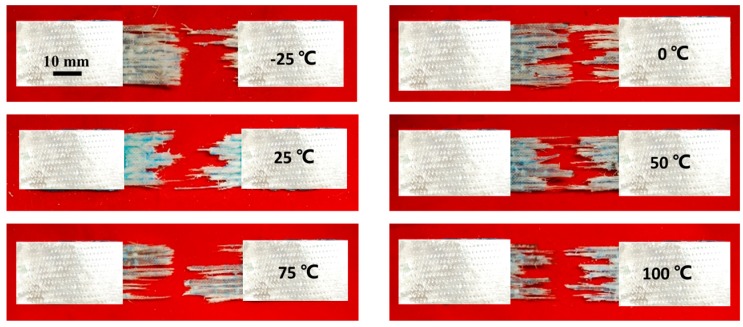
Fracture morphologies of GFRP under different temperatures.

For GFRP, tensile strength shows almost no change (within 3%) when temperature increases from −25 to 50 °C. However, a sharp decrease (about 18.9%) is observed over the temperature range of 50–100 °C, which is caused by the softening of the resin matrix when glass transition temperature of epoxy resin (*T*g) is reached. This softening effect would weaken the interfaces between fibers and matrix, and decrease the stiffness of epoxy matrix during loading. Figure 12 shows the failure morphologies of GFRP specimens at different temperatures. When the testing temperature increases, the components of GFRP specimens are more separated after failure due exactly to the decrease of bonding strength between fibers and matrix. The change of toughness is within 7.6% as temperature increases from −25 to 25 °C, and then decreases as much as 29.2% when the temperature is up to 100 °C. This noticeable decrease is caused by the combined behaviors (decrease) of tensile strength and maximum strain.

## 4. Comparison with Literature

### 4.1. Strain Rate Data

**Figure 13:**
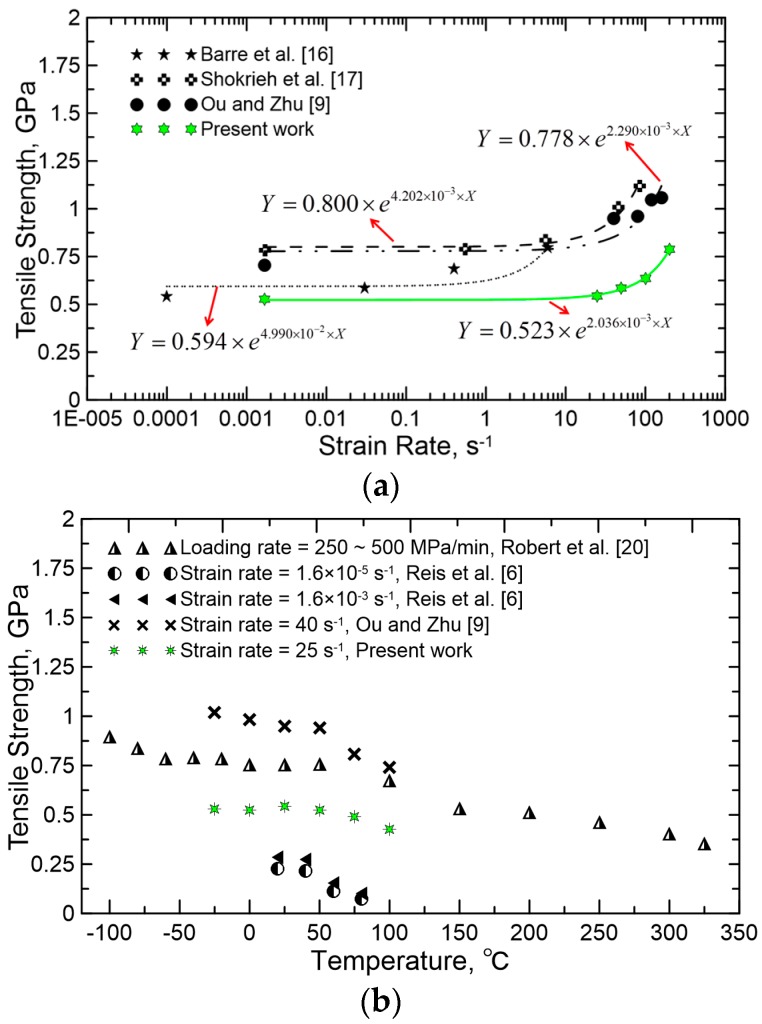
Comparison of the tensile strength of GFRP specimens at different (**a**) strain rates and (**b**) temperatures with literatures.

Figure 13a displays the comparison of the tensile strength of GFRP specimens at different strain rates with literature [9,16,17]. All of the works revealed that the tensile strength of GFRP increases with increasing strain rate, especially over the intermediate strain rate range of 1–200 s^−1^. Assume that tensile strength *TS* is an exponential function of strain rate $\dot{\varepsilon }$ with the following form:

(2) $$TS=A\times e^{\left(B\times \dot{\varepsilon }\right)}$$

Parameters *A* and *B* are then simply determined by a means of exponential fitting, and it proves to be fitting well. Once *A* and *B* are determined, this model can be extrapolated to predict the material behavior at any strain rates within fitting range. In addition, tensile strength of GFRP single yarn reported in previous work [9] is about 150%–200% higher than current results using GFRP samples with eight yarns, indicating a remarkable size effect.

### 4.2. Temperature Data

Figure 13b shows the comparison of the tensile strength of GFRP specimens at different temperatures with the literature [6,9,20]. Note that the tensile strength of GFRP suffers dramatic losses over the temperature range of 50–100 °C, which is caused by the softening of the resin matrix when its glass transition temperature (*T*_g_) is reached. This would weaken the interfaces between fibers and matrices and decrease the resistance of matrices during deformation. Reference [20] also found that the test specimens experienced a significant drop in strength over a temperature range of 300–450 °C. This is due to the thermal degradation of the polymer. In this case, the molecular chains of the polymer broke, leading to the formation of micro-cracks both at the fiber/matrix interface and in the matrix phase [20].

In addition, some researchers [27] proposed that the difference in contractions of fiber and matrix on cooling is also suspected to increase the residual stresses at the fiber/matrix interface, and then result in local micro-cracking and reduced tensile strength. Sánchez-Sáez *et al.* [7] confirmed this when investigating the static behavior of CFRPs at low temperatures. However, reference [20] found that the tensile strength of GFRP increased with decreasing temperature between −100 °C and 0 °C, exhibiting an outstanding capability of resistance to low temperature.

## 5. Weibull Analysis

The determination of the statistics and probability distributions of the random variables characterizing material properties plays an important role in the development of probabilistic-based design specifications. The selection of the probability distribution assigned to characterize the material property data will have a significant effect on the computed reliability. Assuming distinct distributions for the material properties may result in calculated probabilities of failure that vary by over an order of magnitude. This can be attributed to the lower tail behavior of different cumulative distribution functions, which has become acquainted as the tail-sensitivity problem in structural reliability [28]. The Weibull distribution [29] is often used to describe the strength of fibers [30,31] and FRP composites [32,33,34]. Typically, the two-parameter Weibull distribution with the basic form for cumulative probability density is

(3) $$P(\sigma )=1-e^{-{\left(\frac{\sigma }{\sigma_{0}}\right)}^{m}}$$

where σ is the tensile strength, $\sigma_{0}$ is the reference or scaling value related to the mean, and *m* is the Weibull modulus or shape parameter. The cumulative probability density, *P*, is estimated as:

(4) $$P=\frac{i}{N+1},$$

where *N* is the total number of tests and *i* is the current test number.

**Figure 14:**
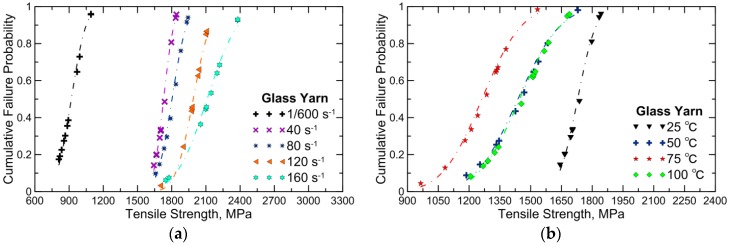

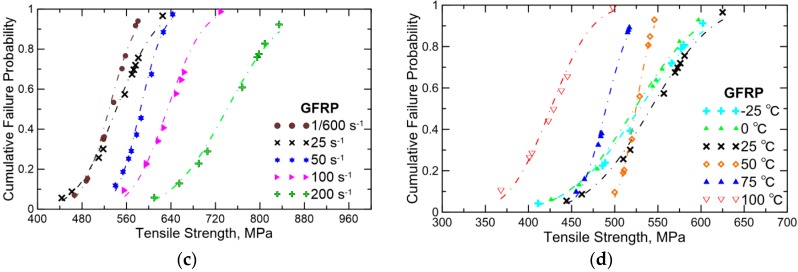
Cumulative failure probability *versus* tensile strength of (**a**,**b**) glass yarn and (**c**,**d**) GFRP under different strain rates and temperatures.

Figure 14 shows the Weibull curve fitting to tensile strengths of glass yarn and GFRP obtained in this work. Figure 14a,c reveal that the cumulative probability plot shifts towards higher values with increasing strain rate, which is a clear indicator of dependence of tensile strength of glass yarn and GFRP on the strain rate. Note that, as shown in Figure 14b, the change of temperatures from 25 to 75 °C result in obvious left-shift of the cumulative probability plot, and then the plot shifts towards the higher stress region when the temperature increases from 75 to 100 °C, in line with Figure 11a. Additionally, as displayed in Figure 14d, no distinct shift of the cumulative probability plot is observed among the temperature range of −25–50 °C. After that, however, the plot remarkably shifts towards the lower stress region, in accordance with Figure 11a as well.

The Weibull parameters extracted from tensile strength data of glass yarn and GFRP samples are presented in Table 2, Table 3, Table 4 and Table 5, respectively. The scale parameter $\sigma_{0}$ generally increases with increasing strain rates, which follows the same change trends of tensile strength at the same testing conditions. Shape parameter *m* is related to the variation of strength among the tested specimens. For glass yarn, in general, the results obtained from higher strain rate have a lower shape parameter than the counterpart. This manifests that yarn subjected to higher strain rate is usually characterized by a more random breakage process.

## 6. Conclusions

This experimental study focuses on the tensile characterization and failure pattern of glass yarn and GFRP samples under different loading conditions. The strain rate and temperature effects on the mechanical properties and fracture morphologies are investigated and discussed comparatively. The following conclusions can be reached:

(1) There is an apparent dependence of the dynamics tensile properties of glass yarn and GFRP on the strain rate. For the glass yarn, tensile strength and toughness increase as much as 88.0% and 474.3% during a transition from quasi-static loading (1/600 s^−1^) to dynamic loading (40 s^−1^), but toughness decreases about 10.2% when the strain rate changes from 120 to 160 s^−1^ due to the diminution of strain. For GFRP, the tensile strength linearly increases nearly 49.1% over the strain rate range of 1/600–200 s^−1^, and toughness increases remarkably (about 109.7%) during a transition from quasi-static loading (1/600 s^−1^) to dynamic loading (25 s^−1^).
(2) The mechanical properties of glass yarn and GFRP are also dependent on the temperature. For glass yarn, tensile strength and toughness decrease nearly 25.3% and 31.1% when temperature increases from 25 to 75 °C. However, when heated to 100 °C, tensile strength and toughness of glass yarn rebound due to the augment of frictional force between fibers. For GFRP, tensile strength shows almost no change (within 3%) when temperature increases from −25 to 50 ° C, but decreases sharply (about 18.9%) over the temperature range of 50–100 °C because of the softening of the resin matrix when Tg of epoxy resin is reached.
(3) The failure patterns of the GFRP specimens are closely related to the loading conditions. At low strain rates, the fracture surface is limited in a small region, while, with increasing strain rate, the damage path covers the entire gauge section where extensive debonding between fibers and matrix was also observed, which leads to an increase in tensile strength and energy absorption. In addition, the components of GFRP specimen are more separated after failure at higher temperatures.
(4) The scale parameter ($\sigma_{0}$) values are highly dependent on the strain rates and temperatures investigated. For glass yarn, the results acquired from higher strain rate have a lower shape parameter than the counterpart. This manifests that yarn subjected to higher strain rate is usually characterized by a more random breakage process.

## Figures and Tables

**Table 1:** Physical and Mechanical Properties of GFRP components (The mechanical properties of glass yarn are obtained under quasi-static loading condition and gauge length of specimen is 25 mm. Those of epoxy resin are provided by manufacturer.).

Components	Tensile Strength, MPa	Young’s Modulus, GPa	Elongation, %	Density, g/cm^3^	C/S Area of Single Yarn, mm^2^
Glass yarn	919	113	1.5	2.54	0.473
Epoxy resin	36	6.1	1.8	1.7	–

**Table 2:** Weibull parameters for tensile strength of glass yarn under different strain rates.

Strain Rate (s^−1^)	1/600	40	80	120	160
*N*	10	8	8	8	8
$\sigma_{0}$ (MPa)	932	1744	1836	2027	2177
*m*	26.1	20.2	16.2	15.4	10.9

**Table 3:** Weibull parameters for tensile strength of glass yarn under different temperatures.

Temperature (°C)	25	50	75	100
*N*	8	8	8	8
$\sigma_{0}$ (MPa)	1744	1479	1312	1497
*m*	20.2	10.3	9.3	8.1

**Table 4:** Weibull parameters for tensile strength of GFRP under different strain rates.

Strain Rate (s^−1^)	1/600	25	50	100	200
*N*	10	10	9	9	9
$\sigma_{0}$ (MPa)	542	560	592	638	766
*m*	12.5	10.1	16.2	15.7	9.2

**Table 5:** Weibull parameters for tensile strength of GFRP under different temperatures.

Temperature (°C)	−25	0	25	50	75	100
*N*	8	8	10	8	8	8
$\sigma_{0}$/MPa	550	541	560	530	496	426
*m*	9.1	8.7	10.1	25.4	18.3	16.0

## References

[1] Bakis C., Bank L.C., Brown V., Cosenza E., Davalos J., Lesko J., Machida A., Rizkalla S., Triantafillou T. (2002). Fiber-reinforced polymer composites for construction-state-of-the-art review. J. Compos. Constr..

[2] Bai Y., Keller T., Vallée T. (2008). Modeling of stiffness of FRP composites under elevated and high temperatures. Compos. Sci. Technol..

[3] Bai Y., Vallée T., Keller T. (2007). Modeling of thermo-physical properties for FRP composites under elevated and high temperature. Compos. Sci. Technol..

[4] Cantwell W.J., Morton J. (1991). The impact resistance of composite materials—A review. Composites.

[5] Kim M., Kang S., Kim C., Kong C. (2007). Tensile response of graphite/epoxy composites at low temperatures. Compos. Struct..

[6] Reis J., Coelho J., Monteiro A., da Costa Mattos H. (2012). Tensile behavior of glass/epoxy laminates at varying strain rates and temperatures. Compos. Part B.

[7] Sánchez-Sáez S., Gómez-del Rıo T., Barbero E., Zaera R., Navarro C. (2002). Static behavior of CFRPs at low temperatures. Compos. Part B.

[8] Zhu D., Rajan S., Mobasher B., Peled A., Mignolet M. (2011). Modal analysis of a servo-hydraulic high speed machine and its application to dynamic tensile testing at an intermediate strain rate. Exp. Mech..

[9] Ou Y., Zhu D. (2015). Tensile behavior of glass fiber reinforced composite at different strain rates and temperatures. Constr. Build. Mater..

[10] Kolsky H. (1949). An investigation of the mechanical properties of materials at very high rates of loading. Proc. Phys. Soc. Lond. Sect. B.

[11] Wang K., Young B., Smith S.T. (2011). Mechanical properties of pultruded carbon fibre-reinforced polymer (CFRP) plates at elevated temperatures. Eng. Struct..

[12] Melin L., Asp L. (1999). Effects of strain rate on transverse tension properties of a carbon/epoxy composite: Studied by moiré photography. Compos. Part A.

[13] Al-Zubaidy H., Zhao X., Al-Mahaidi R. (2013). Mechanical characterisation of the dynamic tensile properties of CFRP sheet and adhesive at medium strain rates. Compos. Struct..

[14] Koerber H., Xavier J., Camanho P.P. (2010). High strain rate characterisation of unidirectional carbon-epoxy im7-8552 in transverse compression and in-plane shear using digital image correlation. Mech. Mater..

[15] Zhou Y., Wang Y., Mallick P. (2004). An experimental study on the tensile behavior of kevlar fiber reinforced aluminum laminates at high strain rates. Mater. Sci. Eng. A.

[16] Barre S., Chotard T., Benzeggagh M. (1996). Comparative study of strain rate effects on mechanical properties of glass fibre-reinforced thermoset matrix composite. Compos. Part A.

[17] Shokrieh M.M., Omidi M.J. (2009). Tension behavior of unidirectional glass/epoxy composites under different strain rates. Compos. Struct..

[18] Ochola R.O., Marcus K., Nurick G.N., Franz T. (2004). Mechanical behavior of glass and carbon fibre reinforced composites at varying strain rates. Compos. Struct..

[19] Hawileh R.A., Abu-Obeidah A., Abdalla J.A., Al-Tamimi A. (2015). Temperature effect on the mechanical properties of carbon, glass and carbon–glass FRP laminates. Constr. Build. Mater..

[20] Robert M., Benmokrane B. (2009). Behavior of GFRP reinforcing bars subjected to extreme temperatures. J. Compos. Constr..

[21] Zhu D., Mobasher B., Rajan S.D. (2011). Dynamic tensile testing of kevlar 49 fabrics. J. Mater. Civ. Eng..

[22] Kuentzer N., Simacek P., Advani S.G., Walsh S. (2007). Correlation of void distribution to vartm manufacturing techniques. Compos. Part A.

[23] Zhu D., Peled A., Mobasher B. (2011). Dynamic tensile testing of fabric–cement composites. Constr. Build. Mater..

[24] Ou Y., Zhu D., Huang M., Li H. (2016). Gage length and strain rate effects on tensile behavior of kevlar 29 single filament and yarn. J. Compos. Mater..

[25] Zhu D., Gencoglu M., Mobasher B. (2009). Low velocity flexural impact behavior of ar glass fabric reinforced cement composites. Cem. Concr. Compos..

[26] Xiao X. (2008). Dynamic tensile testing of plastic materials. Polym. Test..

[27] Reed R., Golda M. (1994). Cryogenic properties of unidirectional composites. Cryogenics.

[28] Ditlevsen O. (1981). Uncertainty Modeling with Applications to Multidimensional Civil Engineering Systems.

[29] Weibull W. (1951). A statistical distribution function of wide applicability. J. Appl. Mech..

[30] Padgett W., Durham S., Mason A. (1995). Weibull analysis of the strength of carbon fibers using linear and power law models for the length effect. J. Compos. Mater..

[31] Hui C., Phoenix S., Shia D. (1998). The single-filament-composite test: A new statistical theory for estimating the interfacial shear strength and weibull parameters for fiber strength. Compos. Sci. Technol..

[32] Alqam M., Bennett R.M., Zureick A.-H. (2002). Three-parameter *vs.* Two-parameter weibull distribution for pultruded composite material properties. Compos. Struct..

[33] Sriramula S., Chryssanthopoulos M.K. (2009). Quantification of uncertainty modelling in stochastic analysis of frp composites. Compos. Part A.

[34] Lekou D., Philippidis T. (2008). Mechanical property variability in frp laminates and its effect on failure prediction. Compos. Part B.

